# Programmed differentiated natural killer cells kill leukemia cells by engaging SLAM family receptors

**DOI:** 10.18632/oncotarget.18659

**Published:** 2017-06-27

**Authors:** Yang Wu, Young Li, Binqing Fu, Linlin Jin, Xiaohu Zheng, Aimei Zhang, Rui Sun, Zhigang Tian, Haiming Wei

**Affiliations:** ^1^ Institute of Immunology and the CAS Key Laboratory of Innate Immunity and Chronic Disease, School of Life Sciences and Medical Center, University of Science and Technology of China, Hefei, China; ^2^ Hefei National Laboratory for Physical Sciences at Microscale, University of Science and Technology of China, Hefei, China; ^3^ Central Laboratory, Anhui Provincial Hospital, Hefei, China

**Keywords:** natural killer cell, programmed differentiation, leukemia, SLAM family receptors and ligands, therapeutic predictor

## Abstract

Natural killer (NK) cells are important innate immune cells that can directly kill transformed or virus-infected cells. The adoptive transfer of NK cells has been used to treat hematological malignancies; however, the limited sources and quantities of NK cells have restricted their clinical application. Here, we acquired sufficient quantities of functional NK cells from CD34^+^ cells treated with a cytokine cocktail. Microarray analysis of the cultured cells revealed a two-stage pattern of programmed differentiation during NK cell development. Different transcription factors were enriched during these two stages, suggesting that preparation of progenitors committed to the NK cell lineage occurs in program 1, while NK cell transformation and maturation occur in program 2. Cultured NK cells highly expressed signaling lymphocytic activation molecule (SLAM) family receptors (SFRs), while leukemia cells expressed SFR ligands. The engagement of these SFRs strengthened the cytotoxicity of NK cells toward leukemia cells. These results demonstrate a simple method of obtaining sufficient NK cells for clinical application, and have categorized NK cell differentiation according to commitment and transformation programs. Moreover, the binding between SFRs on NK cells and their ligands on leukemia cells suggests a new basis for NK cell therapy for treatment of leukemia.

## INTRODUCTION

Natural killer (NK) cells are cytotoxic innate lymphoid cells (ILCs) that are differentiated from T-bet^+^ ILC1, GATA binding protein 3 (GATA-3)^+^ ILC2 and RAR-related orphan receptor gamma (RORγ)t^+^ ILC3 cells [1]. Adoptive NK cell immunotherapy has become increasingly popular because it induces graft-versus-leukemia effects without causing graft-versus-host disease in patients [2]. However, the limited sources and quantities of NK cells still restrict their clinical applications.

The differentiation of NK cells consists of four stages *in vivo*, and is characterized by the surface expression of CD34, CD117 and CD94 [3]. During NK cell development, transcription factors (TFs) promote NK cell commitment and impart corresponding cellular functions. T-bet, Eomesodermin (Eomes), ETS1, inhibitor of DNA binding 2 (ID2), thymocyte selection-associated HMG-box protein (TOX), TOX2, nuclear factor, interleukin 3 regulated (NFIL3/E4BP4) and forkhead box protein O1 (FOXO1) regulate NK cell development and maturation in distinct stages [4–11]. However, the precise hierarchy by which these known TFs regulate NK cell development is not fully understood.

Currently, NK cell activation is believed to be a complex process that integrates hierarchical signals from outside receptors [12]. One important mechanism of NK cell cytotoxicity is their polarized release of lytic granules containing perforin and granzyme toward target cells. The binding of lymphocyte-function-associated antigen-1 to intercellular adhesion molecule-1 can cause granule polarization without cellular degranulation [13]. The processes of polarization and degranulation in NK cells can be uncoupled, and require a strict coordination of signals [14].

Natural cytotoxicity receptors, including NKp30, NKp44, NKp46 and natural-killer group 2, member D (NKG2D), are critical activation receptors in NK cells [15]. Whereas signaling lymphocytic activation molecule (SLAM) cannot be detected on human NK cells, SLAM family receptors (SFRs) including SLAM, NK-, T-, and B-cell antigen (NTBA), CD244, CD84, Ly-9, CD2-like receptor activating cytotoxic cells (CRACC) and CD48 are important co-activating receptors for NK cell function. SFRs are self-ligands that are triggered by homotypic interactions (CD244 and CD48 recognize each other) [16, 17]. Killer inhibitory receptors (KIRs) recognize human leukocyte antigen (HLA) expressed on normal cells to maintain their immune tolerance, a process also known as NK cell licensing. The expression of HLA molecules is often lower in infected or transformed cells than in normal cells, which facilitates NK cell activation [18]. Previous studies have demonstrated that the signals from lymphocyte-function-associated antigen-1 (an indispensable factor), along with any two other activating receptors, are required for successful NK cell cytotoxicity [14]. Thus, the identification of appropriate receptor-ligand interactions is vitally important for effective NK cell activation and clinical applications.

Here, we obtained high-purity NK cells from CD34^+^ cells with a cytokine cocktail. Microarray analysis of the cultured cells suggested that a programmed differentiation pattern occurred during NK cell development. The cytotoxicity of our cultured NK cells was strengthened by the binding between SFRs expressed on the NK cells and their related ligands expressed on primary leukemia cells, suggesting that these molecules could be employed as predictive markers of clinical outcomes for NK cell treatment.

## RESULTS

### Programed differentiation of NK cells from CD34^+^ cells

**Figure 1:**
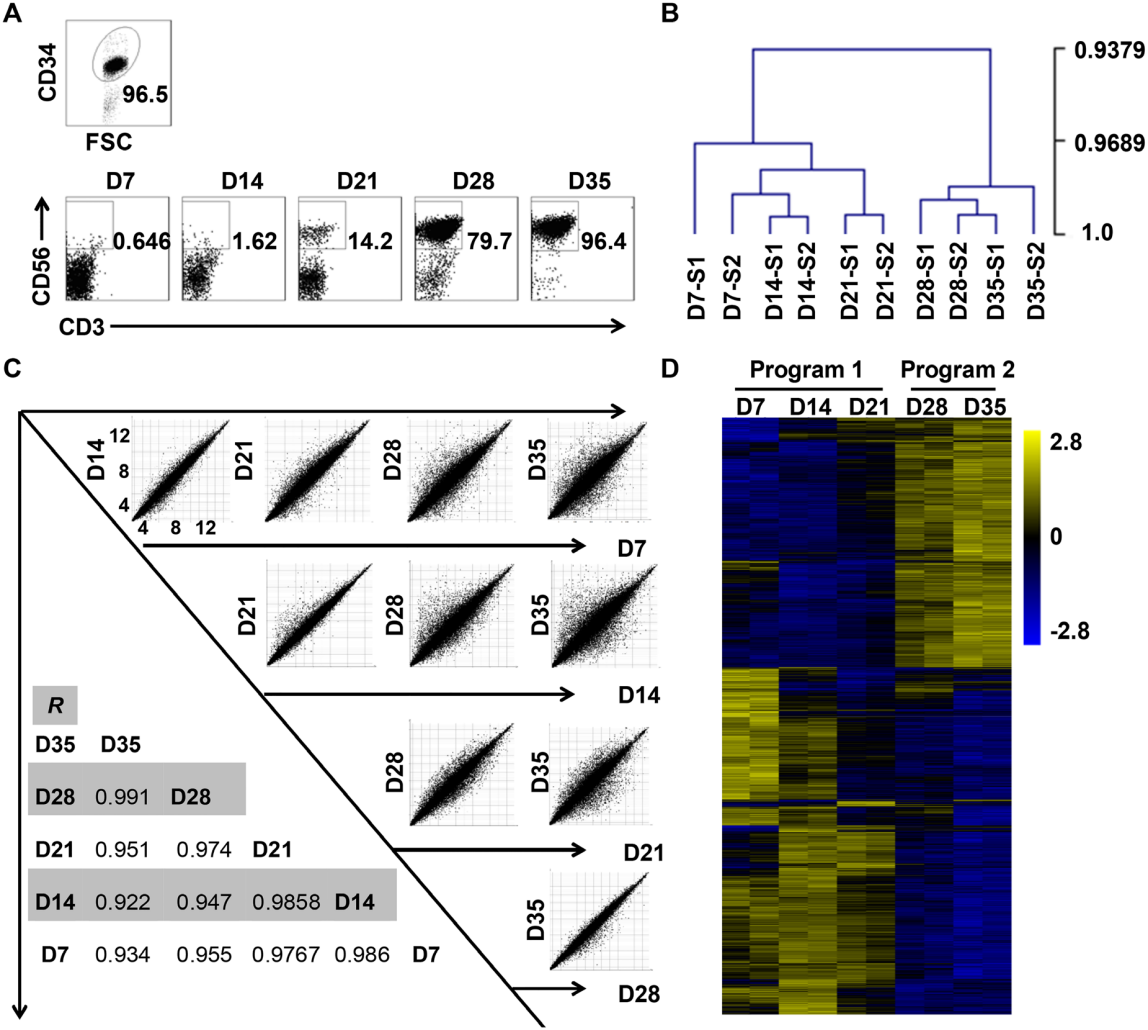
*In-vitro*-differentiated NK cells derived from CD34+ cells can be divided into two developmental programs **(A)** Representative density plots of CD34^+^ cells sorted from UCB mononuclear cells, and flow cytometric analysis of CD56 evaluated on days 7 through 35. **(B)** Hierarchical clustering of cells collected from days 7 through 35, as calculated by MeV 4.9. **(C)** Correlation analysis of cells from each of the two weeks. **(D)** Heat maps displaying differentially expressed mRNA transcripts observed during the culture period (*P* < 0.05).

The flt3 ligand and c-kit ligand can induce the expression of IL-2/15Rβ (CD122) and increase *IL-15Rα* transcripts in CD34^+^ hematopoietic progenitor cells to promote their response to IL-15 [19], which is indispensable for NK cell development and activation [20, 21]. Additionally, IL-21 can induce the maturation and strengthen the function of NK cells [22, 23]. We previously reported that insulin-like growth factor 1 (IGF-1) was critical for human NK cell development and cytotoxicity [24]. Based on these findings, we developed a three-step procedure to obtain sufficient quantities of cytotoxic NK cells from umbilical cord blood (UCB) CD34^+^ cells (Supplementary Figure 1A). In a small-scale culture system, these cells expanded approximately 5000- to 9000-fold (Supplementary Figure 1C). Applying this procedure, we obtained nearly 10^9^ high-quality NK cells at a purity above 95% (Figure 1A & Supplementary Figure 1B, 1D).

We observed the dot plot of NK cells over five weeks, and found that their population sharply increased from less than 15% to nearly 80% from the third week to the fourth week (Figure 1A). Thus, we speculated that *in-vitro*-differentiated NK cells developed at a critical point that primed their explosive development. To explore the cellular biology that occurred during this time course, we conducted a whole-genome microarray analysis on cells collected at different time points from two donors. Notably, correlation analysis of differentially expressed transcripts indicated that the cells clustered more closely during the first three weeks than in the following two weeks (Figure 1C). Furthermore, the hierarchical clustering and heat map of all differentially expressed transcriptomes vividly depicted two distinct stages during NK cell development (Figure 1B, 1D). Given the sharp change in the percentage of NK cells and the results of our microarray analysis, we concluded that NK cells undergo programmed differentiation in two stages *in vitro*. We categorized the first three weeks as program 1 (pre-differentiated cells) and the subsequent two weeks as program 2 (differentiated NK cells).

### Diverse transcription factor classes are enriched during different programs to promote the commitment of differentiated NK cells

**Figure 2:**
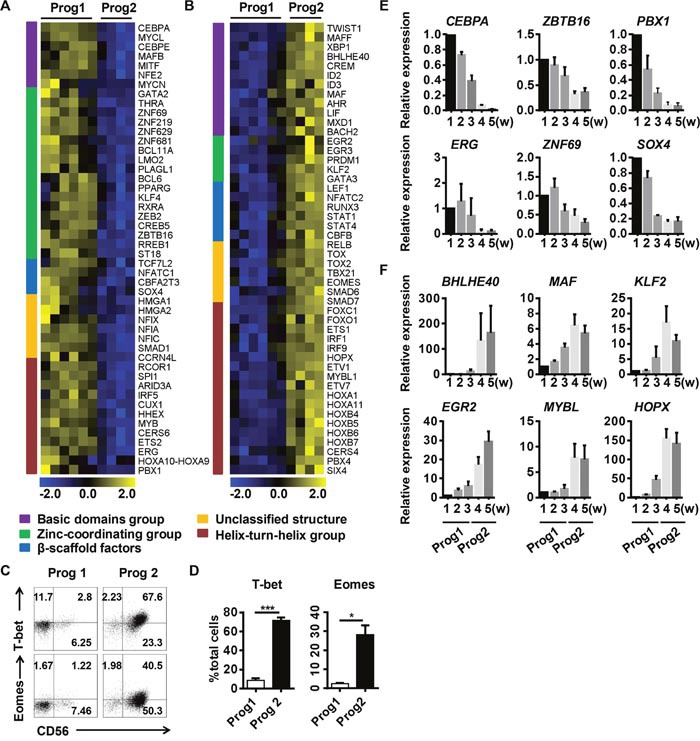
Diverse transcription factor classes are enriched during different programs to promote the commitment of differentiated NK cells Analysis of the different cluster of transcription factor groups expressed at higher levels during **(A)** program 1 or **(B)** program 2. **(C)** Representative density plots of T-bet and Eomes expression analyzed by flow cytometry in gated total cells. **(D)** Quantification of T-bet^+^ or Eomes^+^ cells from gated total cells in program 1 and program 2. **(E, F)** Verification of the transcription factors identified in (A, B) by real-time PCR (n = 4). Results from four samples are presented as the mean ± SEM. *P < 0.05, **P < 0.001 and ***P < 0.0005.

TFs are key determinants of cell growth and differentiation as they bind to regulatory elements in DNA to regulate gene expression [25]. Noting the sharp change in the NK cell percentage during our time course, we focused on the expression pattern of TFs. The expression of established TFs in *in-vitro*-derived NK cells was relatively similar to their expression in peripheral blood (PB) NK cells (Supplementary Figure 2). Furthermore, in flow cytometry, the protein levels of T-bet and Eomes were high in differentiated NK cells during program 2 (Figure 2C-2D), in accordance with previous studies [4, 5, 26]. The similar expression of the established TFs between *in-vitro*-derived NK cells and PB NK cells implied that the former can be used to simulate the development pattern of primary NK cells.

TFs, based on their DNA-binding domains, can be divided into five classes: the basic domain group, the zinc-coordinating group, β-scaffold factors, the helix-turn-helix group, and unclassified structures [27]. We analyzed the differentially expressed TFs in the microchips, and found that cells in program 1 were enriched for zinc-coordinating group TFs (such as *BCL11A* and *ZBTB16*) (Figure 2A, 2E), while cells in program 2 were enriched for helix-turn-helix group TFs (such as *MYBL1*, *HOPX* and *ETS1*) and basic domain group TFs (such as *ID2*, *BHLHE40* and *CREM*) (Figure 2B, 2F). BCL11A is essential for lymphoid development [28], and ZBTB16 (also known as PLZF) is required for the differentiation of ILC progenitors [29]. These results suggested that program 1 is a period for the enrichment of progenitors committed to the NK cell lineage.

Some of the helix-turn-helix family TFs enriched in program 2, such as ETS1, FOXO1, and IRF1, have been shown to regulate NK cell function [6, 11, 30]. HOPX, induced by T-bet, can regulate the persistence of effector memory TH1 cells [31]. Basic region/leucine-zipper motif TFs regulate pathogen defenses in plants, but their functions in mammals have not been demonstrated [32]. BHLHE40 has been shown to function as a cofactor of T-bet that regulates the production of interferon-γ (IFN-γ) [33]. The TFs enriched in program 2 largely promote NK cell maturation and functional competence, suggesting that this program is essential for NK cell transformation and function. The functions of the newly identified TFs in NK cell development warrant further investigation to improve our understanding of the NK cell regulatory network.

In conclusion, we found that TF groups differentially cluster in discrete NK cell development programs. Program 1 prepares progenitor cells for NK cell commitment, while program 2 is responsible for NK cell transformation and functional competence.

### Differentiated NK cells acquire a mature NK cell phenotype

**Figure 3:**
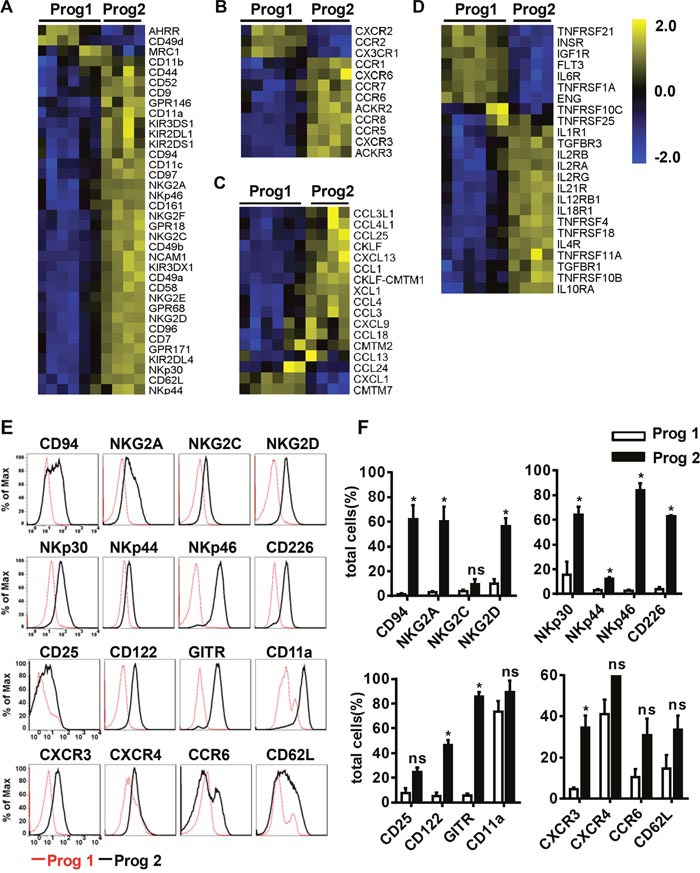
Differentiated NK cells acquire a mature NK cell phenotype **(A, B, C, D)** The variation tendencies of the indicated cell membrane molecules, chemokine receptors, chemokines, and cytokine receptors related to NK cell function. **(E)** Flow cytometric analysis of the expression of the indicated cell membrane molecules measured in program 1 and program 2. **(F)** Quantification of the expressed molecules as a percentage of the total cells. Results from at least three samples are presented as the mean ± SEM. *P < 0.05, **P < 0.001 and ***P < 0.0005.

The integration of signals derived from related receptors is necessary for NK cells to respond to biological changes [12]. In our microarray analysis, we found that the expression of functional membrane molecules closely accompanied the burst of NK cells (Figure 3A). Specifically, the G-protein coupled receptors (GPRs) *GPR18*, *GPR68*, *GPR146* and *GPR171* were upregulated in differentiated NK cells (Figure 3A). As GPRs interact with growth factors, cytokines and chemokines, which are important for NK cell function, their expression by NK cells warrants further investigation [34].

Chemokines can regulate immune cell migration to defend against viral infections or kill transformed cells [35]. We found that differentiated NK cells expressed more chemokine receptors and chemokines than pre-differentiated cells (Figure 3B-3C). It has been reported that activated NK cells secrete CC-chemokine ligand 3 (CCL3) and CCL4, which can augment NK cell cytotoxicity. Additionally, the binding of these chemokines to the CCR5 receptor guides NK cell migration to inflamed tissues [36]. CXCR3 and CCR6, which bind to CXCL9-11 and CCL20, respectively, are also important for NK cell migration [37]. By flow cytometry analysis, we found that NK cell membrane molecules were expressed at higher levels during program 2 than during program 1, with the exception of CXCR4, which was expressed at high levels throughout the entire differentiation process (Figure 3E-3F). Overall, differentiated NK cells obtained a mature NK cell phenotype and the abilities to migrate to abnormal tissues and adhere to transformed cells.

Cytokines are powerful modulators of the immune system, and many of them have been used in the clinic. IL-12, IL-15, and IL-18 enable NK cells to further mature, and induce memory-like functions to strengthen their cytotoxicity toward myeloid leukemia [38, 39]. We found that related cytokine receptors appeared at the appropriate time to promote NK cell differentiation and function (Figure 3D). Cytokine receptors involve in NK cell activation were more highly expressed during program 2 than during program 1, implying that the corresponding cytokines are more useful during that period. These findings suggested that it is important to apply the appropriate cytokines at the proper time to stimulate NK cells for optimal activation and improved cytotoxicity.

### Differentiated NK cells become functional cytolytic effectors

**Figure 4:**
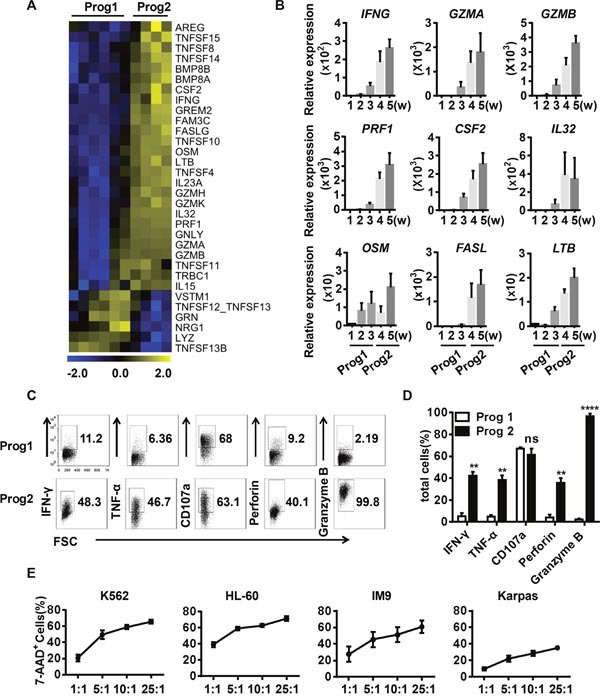
Differentiated NK cells secrete functional cytolytic effectors and efficiently kill target cells **(A)** Heat maps displaying differentially expressed effector molecules in program 1 vs. program 2. **(B)** Verification of the effector molecules identified in (A) by real-time PCR (n = 4). **(C)** Representative density plots of the indicated effector molecules related to NK cell function. **(D)** Percentage analysis of the NK cell molecules expressed in program 1 and program 2 (n = 5). **(E)** The direct cytotoxicity of *in-vitro*-derived NK cells toward K562, HL-60, Jurkat, and Karpas cells, as measured by flow cytometry (n = 5). Results from at least four samples are presented as the mean ± SEM. *P < 0.05, **P < 0.001 and ***P < 0.0005.

NK cells produce various cytotoxic cytokines to kill transformed cells. In contrast to cells in program 1, the cells in program 2 had gene expression profiles that were more related to NK cell function (Figure 4A-4B). For example, IFN-γ, TNF-α, perforin and granzyme B were highly expressed during program 2 (Figure 4C-4D). To verify the cytotoxicity of the differentiated NK cells, we co-cultured NK cells with leukemia cell lines and quantified the 7-Amino-Actinomycin D^+^ (7-AAD^+^) target cells. The differentiated NK cells were clearly more efficient at killing the myeloid leukemia-derived cell lines K562, HL-60 and IM9 than the lymphoid cell line, Karpas-299 (Figure 4E). These data agree with the clinical observation that myeloid leukemia patients are more sensitive to NK cell treatment [2].

### Differentiated NK cells express high levels of SFRs

**Figure 5:**
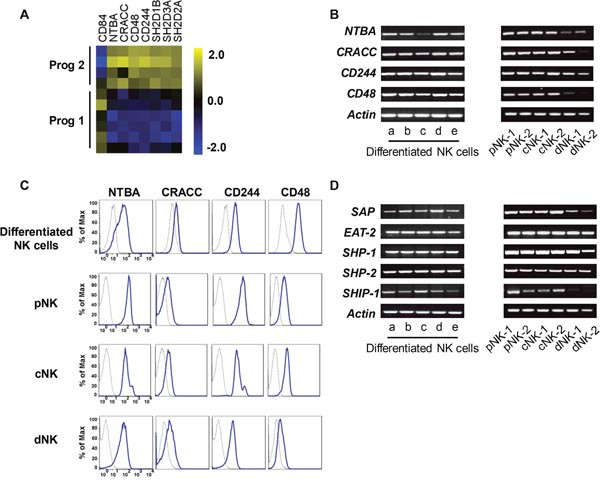
Differentiated NK cells highly express SFRs **(A)** The levels of SFRs revealed by our microarray analysis. **(B)** The detection of SFR mRNA levels from *in-vitro*-derived NK cells, primary PB NK cells, UCB NK cells and decidual NK cells by RT-PCR. **(C)** The protein levels of SFRs from the cells mentioned above. **(D)** The mRNA levels of SLAM-associated protein-related molecules.

NK cell function is not governed by a single activating receptor; instead, the integration of signals regulates their function. Our microarray data indicated that the SFRs were expressed at higher levels during program 2 (Figure 5A). To confirm the data from the microchips, we used RT-PCR and flow cytometry to verify the expression of these genes/proteins in differentiated and primary NK cells. Indeed, differentiated NK cells expressed the four types of SFRs identified in the microchips (Figure 5B-5C). Primary PB NK cells and UCB NK cells clearly expressed nearly all the detected SFRs, whereas decidual NK cells expressed lower levels of these receptors (Figure 5B-5C). The findings suggested a rationale as to why PB NK cells exhibit stronger cytolytic activity than decidual NK cells [24]. Additionally, various NK cell lines exhibited different degrees of SFR expression (Supplementary Figure 3A, 3C).

SFRs contain an immunoreceptor tyrosine-based switch motif, which binds to SLAM-associated protein (SAP)-related molecules including SAP, Ewing's sarcoma activated transcript 2 (EAT-2) and EAT-2-related transducer (ERT) to transduce activating signals. However, ERT cannot be detected in human NK cells [40]. SFRs become activating receptors when they bind to SAP-related adaptors. When these adaptors are not expressed, SFRs become inhibitory receptors by binding to the phosphatases SHIP-1, SHP-1 and SHP-2 [16, 17]. To ascertain whether SFRs serve as activating receptors in differentiated NK cells, we measured the expression of *SAP* and *EAT-2*, and found that both were expressed in program 2, as well as in primary NK cells and NK cell lines (Figure 5D & Supplementary Figure 3B). These findings indicated that SFRs can induce activating signals by binding to SAP-related adaptors in differentiated NK cells.

### Leukemia cells express SFR ligands (SFRLs) at a relatively high level

**Figure 6:**
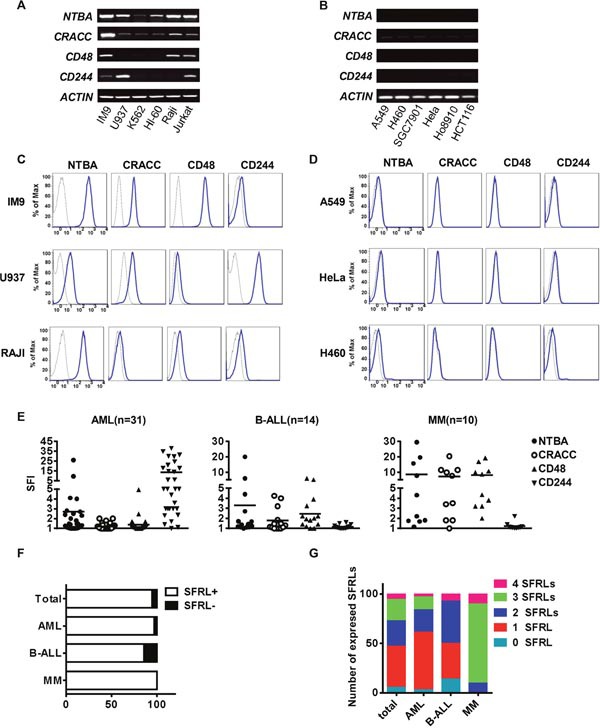
Leukemia cells express relatively high levels of SFRLs The detection of SFRLs in **(A)** leukemia cell lines and **(B)** solid tumor cell lines by RT-PCR. Representative histograms of SFRLs expressed by **(C)** leukemia cell lines and **(D)** solid tumor cell lines. **(E)** The SFIs of various SFRLs detected in individual patients with different leukemia subtypes. **(F)** The proportion of SFRL^+^ (SFI ≥ 1.5 for at least one SFRL) cases among different leukemia subtypes. **(G)** Frequency analysis of leukemia cells in a given leukemia subtype expressing one or more SFRLs.

To identify suitable NK cell targets, we analyzed the expression of SFRLs in different human tumor cell lines. Leukemia cell lines expressed SFRLs at higher levels than solid tumor cell lines (Figure 6A-6D). To determine whether primary leukemia cells express SFRLs, we analyzed bone marrow (BM) cells from leukemia patients. Overall, the expression of at least one SFRL (specific fluorescence index [SFI] ≥1.5) was detected in 52 of the 55 investigated patient cases (Figure 6E). The surface frequencies of expressing at least one SFRL in leukemia cells acquired from acute myeloid leukemia (AML), B-acute lymphoid leukemia (B-ALL) and multiple myeloma (MM) patients were 96.7%, 85.7% and 100%, respectively (Figure 6F). Notably, a certain proportion of leukemia cells acquired from each of these three patient types expressed two or more SFRLs (Figure 6G).

### The engagement of SFRs induces NK cell cytotoxicity toward ligand-positive leukemia cells

**Figure 7:**
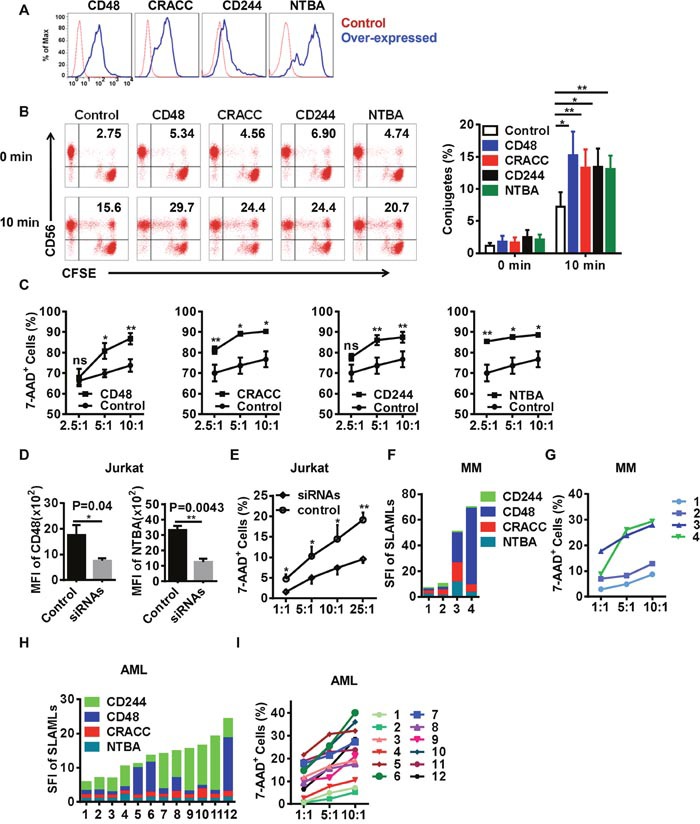
The engagement of SFRs induces NK cell cytotoxicity toward ligand-positive target cells **(A)** Representative histograms of 293T cells overexpressing SFRLs. **(B)** Cultured NK cells were incubated with CFSE-labeled 293T cells with differential SFRL expression. Conjugates were identified as CD56^+^CFSE^+^ cells, and were examined before being co-cultured and after being cultured for 10 min. The left panel displays the representative dot plots examined by flow cytometry, and the right panel graphically represents the conjugates formed at different time points (n = 5). **(C)** The cytotoxicity of differentiated NK cells toward 293T cells overexpressing SFRLs (n = 4). **(D)** The mean fluorescence intensities of CD48 and NTBA in Jurkat cells treated with siRNAs (siCD48 and siNTBA) or control siRNA. **(E)** The cytotoxicity of differentiated NK cells toward Jurkat cells treated with siRNAs (siCD48 and siNTBA) and control siRNA. **(F)** The expression of SFRLs on leukemia cells from MM patients (n=4). **(G)** The cytotoxicity of differentiated NK cells toward leukemia cells from MM patients (n=4). **(H)** The expression of SFRLs on leukemia cells from AML patients (n=12). **(I)** The cytotoxicity of differentiated NK cells toward leukemia cells from AML patients (n=12). Results from at least four samples for the conjugates and cytotoxicity of differentiated NK cells toward 293T and Jurkat cells are presented as the mean ± SEM.*P < 0.05, **P < 0.001 and ***P < 0.0005.

Published reports have demonstrated that SFRs can reinforce immune cell activation [17]. Therefore, we wondered whether SFRs were required for the function of our differentiated NK cells. We transfected 293T cells, which do not express any endogenous SFRs, with *CD48*, *CRACC*, *CD244* and *NTBA*, and used them as target cells (Figure 7A). As conjugate formation is critical for NK cell activation, we measured that between NK cells and the transfected 293T cells. The 293T cells expressing different SFRLs displayed greater conjugate formation than control 293T cells upon co-culture with the differentiated NK cells (Figure 7B). Additionally, the NK cells were more cytotoxic toward SFRL-expressing 293T cells than toward control cells (Figure 7C).

To further determine the effects of SFRs, we used siRNAs to knock down the expression of SFRLs in the leukemia cell lines Jurkat and THP-1, which express high levels of SFRLs (Figure 7D & Supplementary Figure 4A-4B). Differentiated NK cells were less cytotoxic toward cells treated with siRNAs against SFRLs than toward cells treated with control siRNA (Figure 7E & Supplementary Figure 4C).

As NK cell treatments are widely used in leukemia patients, we wondered whether the SFR-SFRL interaction could be used as an evaluation index of the therapeutic effect. Hence, we detected the expression of SFRLs on leukemia cells from MM and AML patients. These cells clearly displayed different SFRL levels and preferences (Figure 7F, 7H). The cytotoxic effects of NK cells toward these leukemia cells were nearly proportional to their SFRL levels (Figure 7G, 7I).

Several other studies have indicated that primary leukemia cells express one or more NKG2D ligands, which bind to NKG2D and thus activate NK cells [41]. As Jurkat cells also express the ligands of NKG2D [42], we wondered whether the binding of these ligands to NKG2D was required for NK cell activation. Indeed, blocking the NKG2D signals of NK cells inhibited their cytotoxicity toward Jurkat cells (Supplementary Figure 4D). The NK cells exhibited the lowest cytotoxic activity when we co-inhibited the signals from NKG2D and SFRs (Supplementary Figure 4D). These results suggested that SFRs and NKG2D synergistically promote NK cell cytotoxicity toward leukemia cells.

Thus, the engagement of SFRs increased the conjugate formation between our differentiated NK cells and their ligand-positive targets, and thereby enhanced the cytotoxicity of the NK cells. Additionally, the differential susceptibilities of leukemia cells to NK cytotoxicity suggested that SFRL levels can be used as clinical predictors of the efficiency of NK cell treatment.

## DISCUSSION

NK cells are innate immune cells that are indispensable for maintaining steady-state physiology. NK cells have been increasingly applied to treat tumors in clinical trials [43–47]. As UCB can be obtained easily and can provide abundant CD34^+^ cells, many researchers have used UCB CD34^+^ cells for NK cell differentiation [23, 48–56]. Importantly, Jan Spanholtz et al. acquired abundant NK cells for clinical application from UCB CD34^+^ cells without feeder cells by adding various cytokines [48]. We developed a procedure to obtain sufficient quantities of functional NK cells with only an essential cytokine cocktail and without feeder cells. The addition of IGF-1, which was found in our previous research to reinforce NK cell cytotoxicity, can further strengthen NK cell anti-leukemia activity [24]. We acquired similar or higher yields of NK cells with 5% human AB type serum, ensuring that the NK cells could be used in the clinic (data not shown). Furthermore, our differentiated NK cells expressed high levels of CXCR4, which could guide these cells to eliminate leukemia cells residing in the BM [35]. The production of high levels of cytokines and chemokines makes NK cells more powerful and may attract other immune cells, allowing them to function together. We determined that differentiated NK cells expressed high levels of SFRs. The specific expression of SFRLs on leukemia cells, not on solid tumor cells, offers an important mechanism by which NK cells can be used to treat leukemia.

Previous reports have identified four discrete stages in the *in vivo* transition from a CD34^+^ hematopoietic progenitor to a functional NK cell [3]. However, researchers have never described the pattern of changes in developing NK cells. In our microchip analysis, we noticed that the differentially expressed transcripts changed dramatically alongside a change in NK cells. With a hierarchical clustering algorithm and correlation analysis, we detected a clear two-program pattern of development during NK cell differentiation. The differential clustering of TFs suggested that program 1 prepares progenitors for commitment to the NK cell lineage, while program 2 fosters NK cell transformation and maturation. We believe that one or more TFs are critical for the rapid conversion of NK cells; this warrants further study.

NK cell activation requires the integration of signals from activating and inhibitory receptors [12, 57, 58]. Our cultured NK cells express numerous activating receptors (NKp46, NKp30, and NKG2D) and co-activating receptors (SFRs) to ensure their function. In addition, it has been reported that the high expression of CD11a by NK cells allows them to form an immunological synapse and thus seize their targets [13]. Researchers have already done tremendous work investigating the regulation of NK cell activity by SFRs and SAP-related adaptors [17]. In our study, we found that leukemia cell lines and primary leukemia cells highly expressed SFRLs, while none of the solid tumor cells did. Murine hematopoietic malignancy cells exhibit a similar phenomenon [40]. We determined that the SFR-SFRL binding between NK cells and their target cells strengthened their conjugate formation and enhanced the cytotoxicity of the NK cells. Furthermore, differentiated NK cells displayed stronger cytotoxicity toward primary leukemia cells expressing higher levels of SFRLs. These results implied that leukemia cells may be more susceptible to NK cells than other cancer cells, and that SFRL levels can be used as clinical predictors of the efficiency of NK cell treatment. Previous studies have demonstrated that NK cells can kill leukemia cells by binding to their NKG2D ligands with NKG2D [41]. The high expression of NKG2D and SFRs by our differentiated NK cells could allow them kill their target leukemia cells more effectively.

Because leukemia stem cells, which reside in the BM, are the leading cause of relapse, we are convinced that treatments targeting the BM would eliminate any minimal residual disease. Importantly, our differentiated NK cells consistently expressed the chemokine receptor CXCR4, which is important for the homing of immune cells to the BM [35]. Additionally, *in-vitro*-derived CXCR4^+^ NK cells have been shown to migrate to the BM in murine models of leukemia [37]. T cells have also been shown to traffic to the BM efficiently via the CXCR4/CXCL12 axis in prostate cancer patients [59], which implies that adoptively transferred NK cells may also be able to traffic to the BM in leukemia patients. Interestingly, their high chemokine expression enabled our differentiated NK cells to attract other immune cells for coordinated functions.

In conclusion, we developed a procedure whereby sufficient quantities of NK cells can be generated with a minimal cytokine cocktail. Our differentiated NK cells were functional effectors that highly expressed cytotoxic cytokines and were highly cytotoxic toward tumor cells. Moreover, we identified a programmed differentiation pattern that occurs during NK cell development, and provided a platform for studying its transcriptional regulation and novel molecules important for its function. In addition, we identified SFR-SFRL binding between NK cells and leukemia cells, supporting adoptive NK cell therapy for hematological malignancies. SFRL expression on leukemia cells could also serve as a predictor of clinical outcomes for NK cell therapy.

## MATERIALS AND METHODS

### Samples

UCB, PB, decidual and clinical leukemia patient samples were obtained after informed consent was provided. The clinical characteristics of the leukemia patients are presented in Supplementary Table 1. Approval was obtained by the Ethics Committee of the University of Science and Technology of China.

### Differentiation of NK cells from UCB CD34^+^ cells

UCB CD34^+^ cells were freshly isolated from mononuclear cells with magnetic-activated cell sorting (MACS; Miltenyi Biotec, Bergisch Gladbach, Germany). Cells were cultured in a 1:1 α-MEM (Hyclone): Serum-free Stem Cell Growth Medium (CellGro) mixture supplemented with 10% fetal bovine serum (Gibco) in a humidified atmosphere of 5% CO_2_ at 37°C. For the detailed procedure, see Supplementary Figure 1A. The cytokines were purchased from PeproTech. Half of the culture medium was replaced twice per week with fresh medium.

### Flow cytometry

Single-cell suspensions were labeled with fluorochrome-conjugated human monoclonal antibodies (mAbs) for the analysis of cell membrane and intracellular molecules. For details about the mAbs, see the Supplemental Materials. Stained cells were detected on an LSR II flow cytometer (BD Biosciences) and analyzed with FlowJo software (Tree Star). The specific fluorescence indices (SFI) were calculated as follows: median fluorescence of the respective specific mAb/median fluorescence of the isotype control [41].

### Microarray analysis

Microarray analyses were performed at different time points with PrimeView chips (Affymetrix) with cultured cells obtained from two donors. The hierarchical clustering algorithm and heat map of differentially expressed genes were analyzed with MeV 4.9 software. The microarray data were deposited into the GEO repository under accession number GSE87787.

### Carboxyfluorescein succinimidyl ester (CFSE)-based cytotoxicity assay

The cytotoxic activity of NK cells was determined in a CFSE-based cytotoxicity assay [60]. For details, see the Supplemental Methods. The percentage of cytotoxicity was calculated as follows: % cytotoxicity = 100 × (experimental release-spontaneous release)/(100-spontaneous release).

### Transduction and conjugate formation

*CD48*, *CRACC*, *CD244* and *NTBA* were cloned into the PCDH-CMV-MCS-EF1- puro vector (Addgene). Then, 293T cells were transfected with the plasmids via Lipofectamine 2000 (Invitrogen), according to the manufacturer's protocol. On the second day, the transfected cells were co-cultured with NK cells and analyzed for conjugate formation and susceptibility to NK cell cytotoxicity. The conjugate formation assay was performed as described previously [61]. Briefly, NK cells were labeled with a CD56 mAb and resuspended in phosphate-buffered saline. The 293T cells were labeled with CFSE as described above. Then, the NK cells and 293T cells were incubated at 37°C for different time intervals, and ice-cold phosphate-buffered saline was added to stop the reactions. The conjugates, defined as CD56^+^CFSE^+^, were analyzed by flow cytometry.

### Nucleofection

The siRNAs against human *NTBA*, *CRACC*, *CD244* and *CD48*, along with siRNA mimics, were purchased from Santa Cruz. The Amaxa Cell Line Nucleofector Kit V (VCA-1003) was used to transfect Jurkat and THP-1 cells with the X-001 and V-001 programs, respectively. Cells were plated in complete RPMI 1640 medium, and NK cell cytotoxicity was detected.

### Statistical analysis

Data are presented as the mean ± SEM generated by GraphPad Prism. The data were analyzed with paired or unpaired two-tailed *t*-tests and one-way ANOVA. *P* values less than 0.05 were considered to indicate statistical significance.

## SUPPLEMENTARY MATERIALS


