# Supplementary material for: Programmed differentiated natural killer cells kill leukemia cells by engaging SLAM family receptors
Source: Oncotarget. 2017 Jun 27;8(34):57024–38. doi: 10.18632/oncotarget.18659 (PMC5593622; doi:10.18632/oncotarget.18659)
Supplement: Supplementary file 1 [file oncotarget-08-57024-s001.pdf]

Programmed differentiated natural killer cells kill leukemia cells by engaging SLAM family receptors

SUPPLEMENTARY MATERIALS

Samples

Cord blood samples were collected at birth after normal, full-term delivery at Anhui Provincial Hospital and the Maternity and Child Care Hospital, Hefei. Decidual samples were obtained from normal donors with elective pregnancy terminations at the Maternity and Child Care Hospital, Hefei. Human PB samples were obtained at Hefei Blood Bank. BM samples were obtained from leukemia patients at the time of diagnosis and prior to therapy at Anhui Provincial Hospital.

Antibodies

The following antibodies were purchased from BD Pharmingen: CD34, CD3, CD56, CD48, CD226, NKp30, NKp44, NKp46, CD94, NKG2C, NKG2D, CD11a, CD62L, CD25, CD122, CD244, CXCR3, CCR6, IFN-γ, TNF-α, perforin, granzyme B, and CD107. NTBA, CRACC and NKG2A were purchased from R&D Systems; T-bet, Eomes and CXCR4 were purchased from eBioscience. Isotype-matched antibodies were all obtained from BD Pharmingen. The purified anti-human NKG2D antibody and IgG1 isotype control were purchased from Biolegend.

Cell lines

A549 (human lung carcinoma), H460 (large cell lung cancer), SGC7901 (human gastric adenocarcinoma), HeLa (cervical cancer), HO8910 (human ovarian carcinoma), HCT116 (epithelial colon colorectal carcinoma), K562 (erythroleukemia), HL60 (acute promyelocytic leukemia), Raji (Burkitt lymphoma), Jurkat (acute T cell leukemia) and THP-1 (acute monocytic leukemia) cells were purchased from the Shanghai Cell Bank (Chinese Academy of Sciences, Shanghai, China). Karpas-299 (T-cell lymphoma) cells were purchased from DSMZ (Germany). IM9 (multiple myeloma), U937 (histiocytic lymphoma) and NK92 (NK cell line) cells were purchased from the American Type Culture Collection (USA). YT (NK-like cell line) cells were obtained from the NCI (USA). NKL (NK cell line) cells were kindly provided by Professor BQ Jin (Fourth Military Medical University, Xi 'an, PR China). NKG (NK cell line) cells were established in our lab (Department of Immunology, University of Science and Technology of China, Hefei).

RNA isolation and quantitative real-time PCR

Total RNA was extracted with TRIzol reagent (Invitrogen) according to the manufacturer's instructions. First-strand cDNA was synthesized with Moloney murine leukemia virus reverse transcriptase (Invitrogen) and oligo $(dT)_{20}$ primers. Then, quantitative real-time PCR was performed with SYBR Premix Ex Taq (TaKaRa) with primers for the relevant target transcripts. The mRNA level of *β-actin* was used as a normalization control. The data were analyzed with the $2^{-\Delta\Delta Ct}$ method.

The gene-specific primers are shown in Supplementary Table 2.

CFSE-based cytotoxicity assay

Briefly, target cells were labeled with 5 μM CFSE (Sigma) for 15 min at 37°C. After being labeled, the cells were resuspended in RPMI 1640 medium (Hyclone). Effector cells and target cells were added to 96-well plates at different effector-target ratios for 6 hours at 37°C and 5% CO_2. For the detection of dead cells, 7-AAD (BD Pharmingen) was added, and the samples were directly analyzed by flow cytometry.

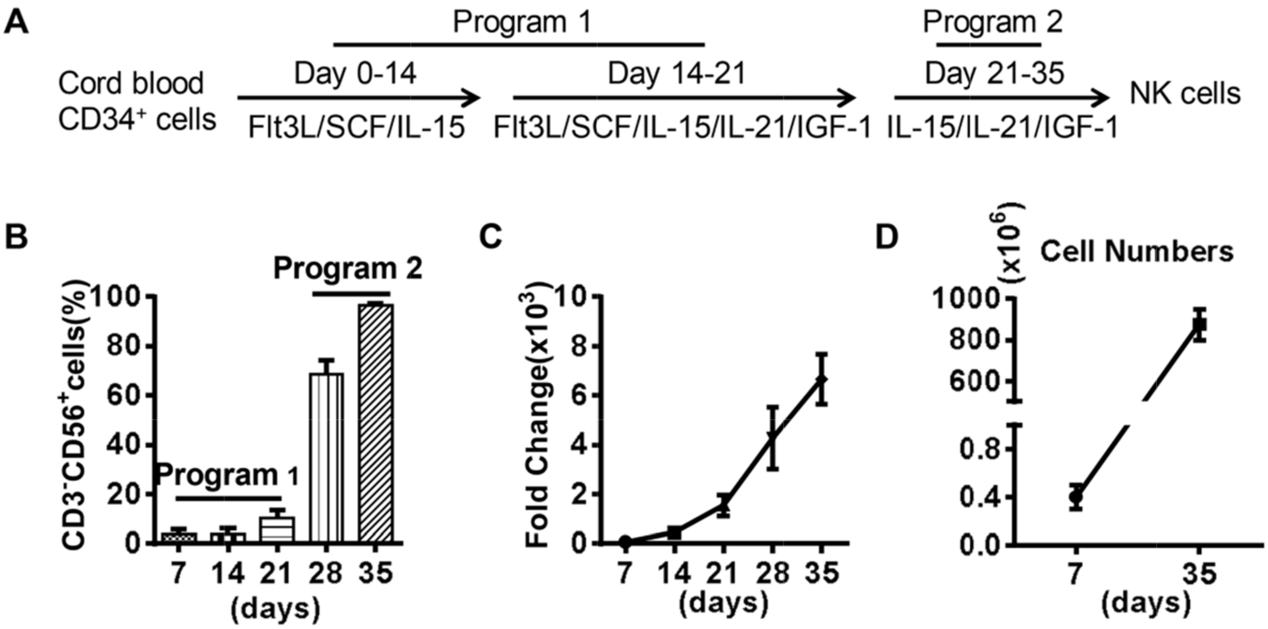

Supplementary Figure 1: NK cells differentiated from cord blood CD34+ cells. **(A)** Flow diagram of the culture method for differentiating and expanding NK cells from cord blood CD34^{+} cells cultured with a cytokine cocktail. **(B)** The frequency of CD56^{+}CD3^{-} cells as a proportion of the total gated cells during a 35-day time course. **(C)** The fold-change of cultured cells over the 35-day time course. **(D)** The total cell numbers calculated from the beginning to the end of the culture.

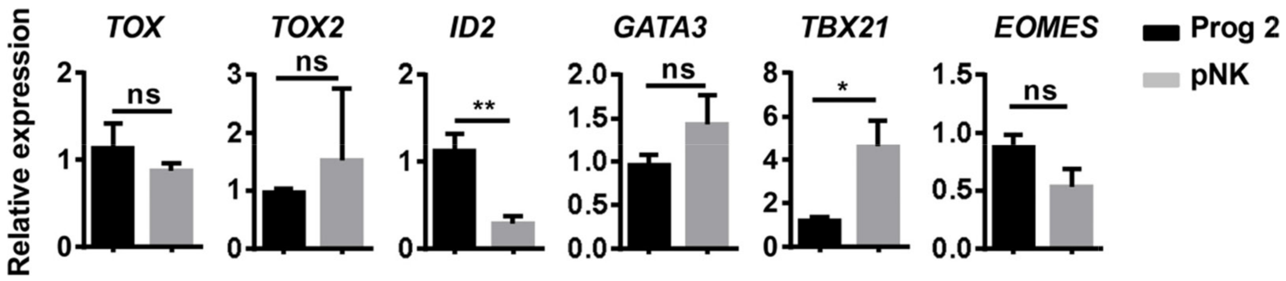

Supplementary Figure 2: The detection of TFs known to function during NK cell development. The expression patterns of established TFs were evaluated in *in-vitro* derived-NK cells and PB NK cells. Results from four samples are presented as the mean ± SEM. $*P < 0.05$, $**P < 0.001$ and $***P < 0.0005$.

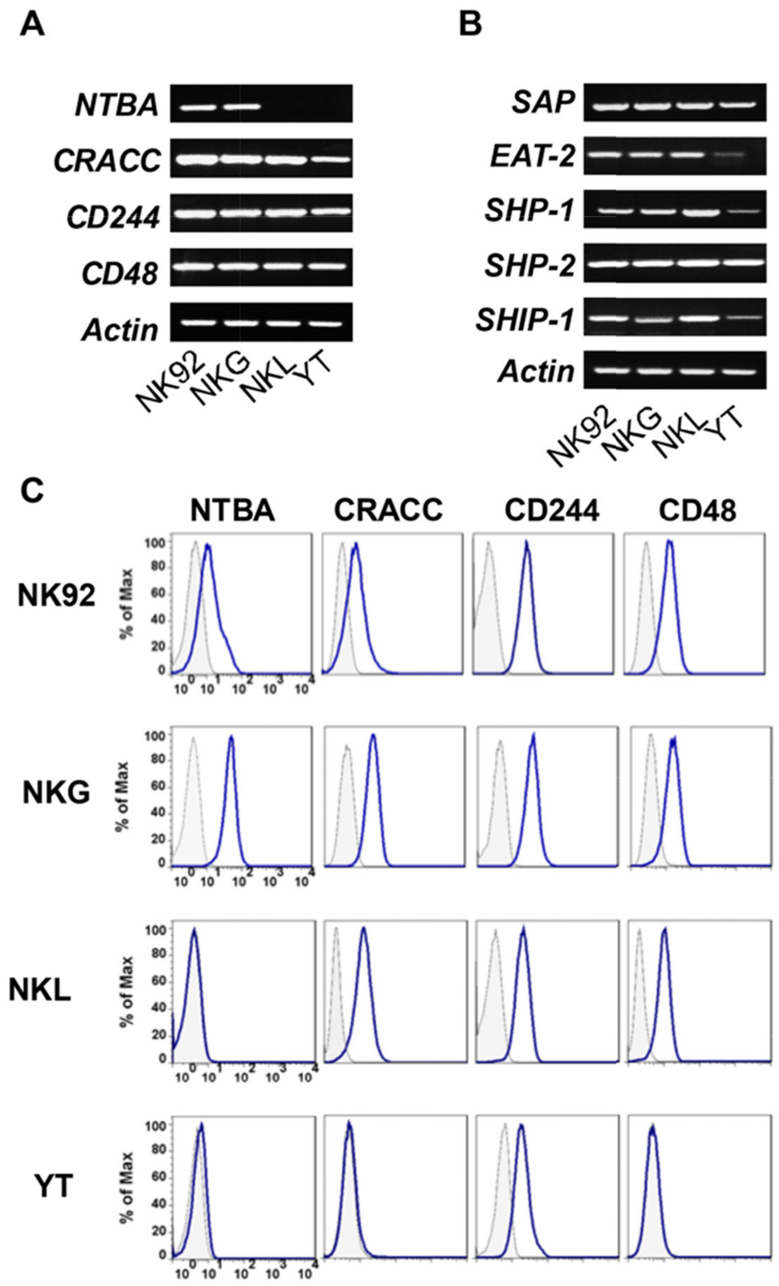

Supplementary Figure 3: The expression patterns of SFRs in NK cell lines. **(A)** The detection of SFRs and **(B)** SLAM-associated protein-related molecules expressed in NK cell lines by RT-PCR. **(C)** Representative histograms of SFRs expressed by NK cell lines, as measured by flow cytometry.

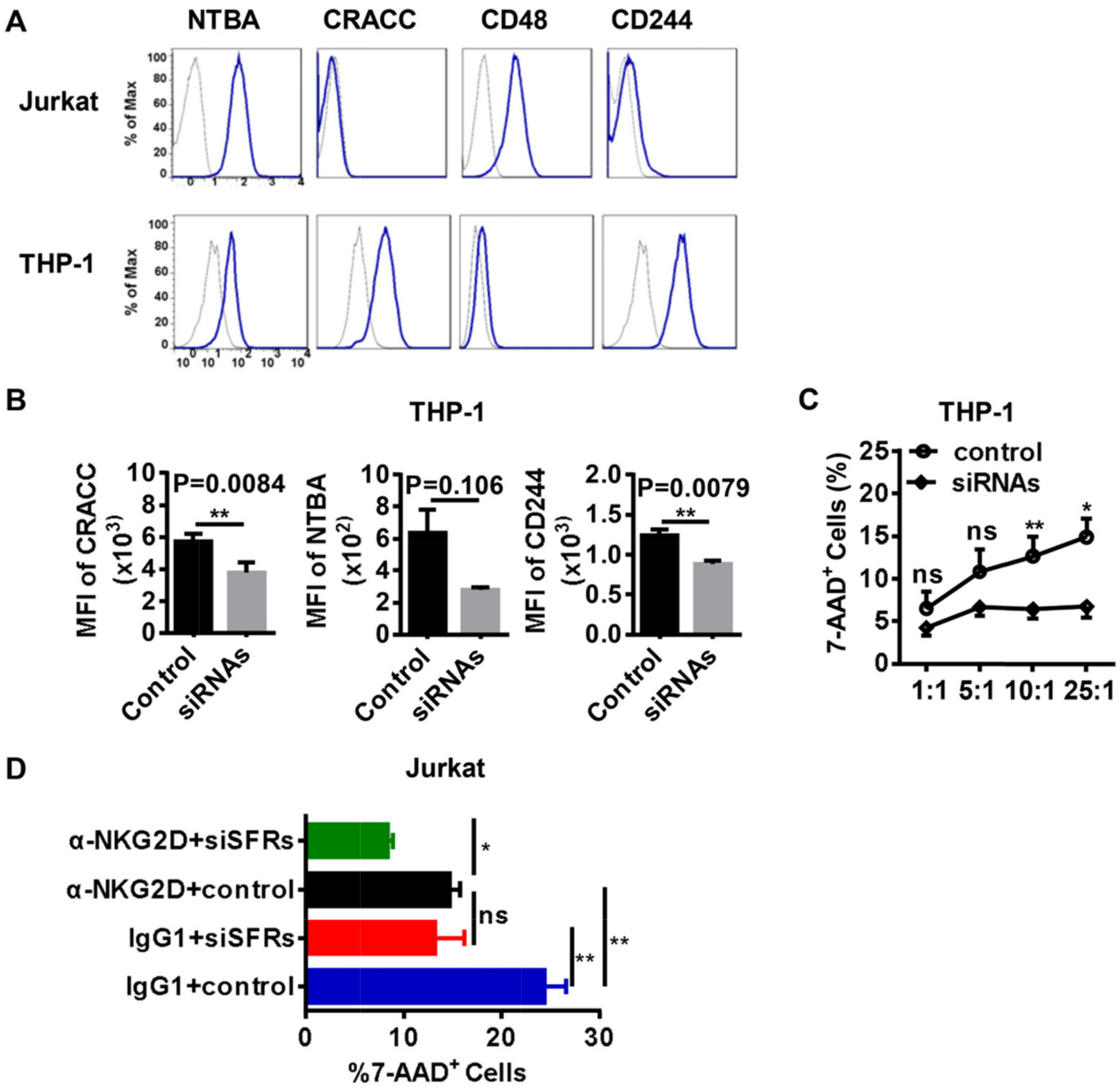

Supplementary Figure 4: Knock-down of SFRL expression reduced the cytotoxicity of NK cells. **(A)** Representative histograms of SFRLs expressed by Jurkat and THP-1 leukemia cell lines. **(B)** The mean fluorescence intensities of CRACC, NTBA and CD244 in THP-1 cells treated with siRNAs (siCRACC, siNTBA, and siCD244) and control siRNA. **(C)** The cytotoxicity of differentiated NK cells toward THP-1 cells treated with siRNAs and control siRNA (n=6). **(D)** Cytolytic assays of NK cells were performed in the presence of an NKG2D antibody or siSFRs (siCD48 and siNTBA), used alone or in combination (n=4). The E/T ratio used was 15:1. Results from at least four samples are presented as the mean ± SEM. $^*P < 0.05$, $^{**}P < 0.001$ and $^{***}P < 0.0005$.

Supplementary Table 1: Clinical characteristics of leukemia patients

See Supplementary File 1

Supplementary Table 2: The gene-specific primers

Gene	Sequence (5'-3')	Gene	Sequence (5'-3')
CEBPA-F	TATAGGCTGGGCTTCCCCTT	*IFNG-F*	TGTCGCCAGCAGCTAAAACA
CEBPA-R	AGCTTTCTGGTGTGACTCGG	*IFNG-R*	TGCAGGCAGGACAACCATTA
ZBTB16-F	TGGGGTCGAGCTTCCTGATA	*GZMA-F*	ATCTGTGCTGGGGCTTTGAT
ZBTB16-R	GGCCATGTCAGTGCCAGTAT	*GZMA-R*	TGCTTTTTCCGTCAGCTGTAA
PBX1-F	AACTCGGCTGGTTCTTCCAG	*GZMB-F*	CCCTGGGAAAACACTCACACA
PBX1-R	TCCCCATTGAGTGACTGCAC	*GZMB-R*	CACAACTCAATGGTACTGTCGT
ERG-F	TTCACTTGGTCGGAATGGGG	*PRF1-F*	CGCCTACCTCAGGCTTATCTC
ERG-R	CTGACGGCTTTAGTTGCCCT	*PRF1-R*	CCTCGACAGTCAGGCAGTC
ZNF69-F	GCTGTAGTCACAGGAGGTGT	*CSF2-F*	AATGTTTGACCTCCAGGAGCC
ZNF69-R	GAGACTCCTGAAGTTTCTCCTGG	*CSF2-R*	CCCTTGGTCCCTCCAAGATG
SOX4-F	CAGCAAACCAACAATGCCGA	*IL32-F*	CGTGTGACACTGAGGACACT
SOX4-R	GATCTGCGACCACACCATGA	*IL32-R*	CGACATCACCTGTCCACGTC
BHLHE40-F	GAAGCTGCATCTCAAAGCCG	*OSM-F*	AGTGGGGACATGGACTGGAA
BHLHE40-R	GGGTACATCCCTGGTAGGTCT	*OSM-R*	TACGTATATAGGGGTCCAGGAGTC
MAF-F	AGCAAGTCGACCACCTCAAG	*FASL-F*	CCTTGGTAGGATTGGGCCTG
MAF-R	CTGGAATCGCGTGTCAGACT	*FASL-R*	TCTGGCTGGTAGACTCTCGG
KLF2-F	CACACAGGTGAGAAGCCCTACC	*LTB-F*	GGAGCCACTTCTCTGGTGAC
KLF2-R	TCGTCAAGGAGGATCGTGGT	*LTB-R*	TCTGAAACCCAGTCCTCCCT
EGR2-F	GGCTTTTCTGACACTCCAGGTA	*NTBA-F*	TTTGGCCCAGGACAACTGAGG
EGR2-R	TCCAACGACCTCTTCTCTCCA	*NTBA-R*	TGCAGGTGTAGTCCTGTTCAC
MYBL-F	AAAGCGAATTCCATCACAGCC	*CRACC-F*	CTGGTCGGTTCCGTTGG
MYBL-R	TGGGATGGTCTGCAAAGAGG	*CRACC-R*	ACTTTAGGCTTTGACAGGTGC
HOPX-F	GAGGAGACCCAGGGTAGTGAT	*CD48-F*	ACTGCTGCCTCTGTCACTCC
HOPX-R	TCTGTGACGGATCTGCACTC	*CD48-R*	TCTTGCTCATTCCCAGTCTTT
ETS1-F	CGATCTGGAGCTTTTCCCC	*CD244-F*	GGGAGAATGGCTCTTTGCCT
ETS1-R	TGGAGTTAATAGTGGGACATCTGC	*CD244-R*	CGGGGTTTCTCAACTTTATCAAATA
ID2-F	TGAAAGCCTTCAGTCCCGTG	*SAP-F*	AAGTACACAAGGTACTACAGGGAT
ID2-R	AGGGAATTCAGAAGCCTGCAA	*SAP-R*	AAGGGACACCAGCCAACTTC
TBX21-F	CACGTCCACAAACATCCTGT	*EAT-2-F*	GACAGCGAGTCGATACCAGG
TBX21-R	GATCATCACCAAGCAGGGAC	*EAT-2-R*	CCTTCTGCAGTCTGTATCCTGT
TOX-F	AACCGGTGGACTGGAATAAC	*SHP-1-F*	TTCGGATCCAGAACTCAGG
TOX-R	TGGAGAACTGCCTTGACTGT	*SHP-1-R*	TGCAAACTCTCAAACTCCTC
TOX2-F	CACCTGGACTATTACCACGGC	*SHP-2-F*	GGAGCTGTCACCCACATCAA
TOX2-R	TTGTAGGTCTGGCTGGTTGAC	*SHP-2-R*	CCGTCATTGCTCTCCCCTTT
EOMES-F	CTGGCTTCCGTGCCCACGTC	*SHIP-1-F*	AAGTGGCCACAACTCTCCTG
EOMES-R	CATGCGCCTGCCCTGTTTCG	*SHIP-1-R*	CGCAATTCCGATACAGCACG
ACTIN-F	TTGCCGACAGGATGCAGAA		
ACTIN-R	GCCGATCCACACGGAGTACTT		

F, Forward. R, Reverse.
